# Technique for the Double Grip, Unilateral Cases and Short Double Abutment, Cast Clasp Bridges

**Published:** 1920-12

**Authors:** F. Ewing Roach

**Affiliations:** Chicago, Ill.


### TECHNIQUE FOR THE DOUBLE GRIP, UNILATERAL CASES AND SHORT DOUBLE ABUTMENT, CAST CLASP BRIDGES

BY DR. F. EWING ROACH, CHICAGO, ILL.

Before taking up the technique of the two types of cases we have to consider this morning, I will refer briefly again to the necessity for care in handling these alloys for cast clasps. I am satisfied that these metals are being abused. Alloys of platinum, palladium and gold contain copper. If you heat them too hot, you are sure to materially injure the alloy and may even ruin it. It will become brittle and overheating it will make it cast poorly. It will be sluggish; the oxidation of the copper will produce this condition. Use a low heat flame. If using nitrous oxid, or oxygen and gas, presto-lite or any of those high heat concentrated flames, you are running a great risk. Melt the gold carefully. Another point with reference to refining these alloys. You will always have a surplus and you can use the excess any time you want to use the material previously cast; but it is necessary to clean or refine the material before casting it again. In the casting process you are sure to have contamination of the silica that is incorporated in the investment compound, as it always carries a little iron. A very small amount of iron will injure the alloy. These alloys, when being used the second or more times, should be cleaned before re-casting. You can clean them by placing them in hydrofluoric acid, or better, by cleaning the silica off mechanically; i. e., by having a small dish-shaped piece of fire clay that you can lay the gold on; cover the surface with borax, heating until you have a glassy surface over the fire clay.

* Lecture before the Southern California Dental Association, Los Angeles, July 2, 1919. Reprinted from *Pacific Dental Gazette*

Lay the gold on this fire clay and bring to a red heat. Throw borax over the surface and as it melts the borax will unite with the silica and as the pellet of gold is in a molten condition, you will see the scum disappear from the surface. After the gold is melted, pour it off, mechanically removing the silica from the gold. I have had the manufacturers of these golds verify this method. Now it is also important that you do not use saltpeter in refining these alloys; simply resort to the mechanical means I have referred to. These alloys, when handled properly, are very tough and strong. If you get brittleness and breakage of clasps I am inclined to think it is due to faulty manipulation, rather than to defective material.

I will go through the technique of making the double clasp short span removable bridge, where we have two sound teeth on both sides of the space. It is not necessary to revert to the reasons for supplying even one tooth, molar or bicuspid. Of course, if it is an anterior tooth, possibly a first bicuspid, most people are too particular about their personal appearance to allow the space to remain, and will have something done. But it is a very common occurrence for our patients to leave spaces in the posterior part of the mouth when only a single tooth has been extracted—the loss of the first molar particularly. It is responsible for a great amount of damage, and we have been in the habit of leaving them alone, rather than mutilate the teeth on both sides of the space and put in a fixed bridge or plate to supply these missing teeth. Thus we have believed we are doing less damage by leaving them untouched rather than undertaking to supply some artificial substitute for the lost tooth. And, considering the necessities of fixed bridgework construction, probably we have done the right thing. But it is my belief at this time that with the simplified technique and the possibilities of the cast clasp, we can meet the requirements of most of these cases, if we put them in immediately or shortly after the extraction of the natural teeth, supplying a cast removable bridge without any material amount of mutilation of the natural teeth, thus giving our patients something entirely comfortable and useful. There is no question at all about the damage that occurs from the loss of one tooth or more—the loss of normal occlusion. The loss of proper contact and occlusion are responsible for possibly more decay, more pyorrhea and the loss of more teeth than any one thing. I will give you what in my judgment is a very simple and definite technique for an appliance that is useful and will maintain the normal relations of the teeth. I find many cases of this type, as almost every mouth you examine will show that a molar or bicuspid is missing. If teeth have been out a long time and the abnormal conditions have already taken place, it is a question whether we can do the patient any particular benefit by putting in the appliance. Our duty is to put these pieces in at the time of extraction and before the damage occurs. But there are many cases in which there is a missing tooth or two, that may be greatly benefited by the placement of an appliance of this kind—in cases in which the occlusal conditions and relations are practically normal, and sometimes for cosmetic reasons, it may be necessary to supply these appliances.

These short cases require more careful and exact construction measures than the large cases. The one-tooth case is more difficult than the two-tooth case, because of the short span. I made these appliances as I did with the larger ones for a long time; I made impression of the ridges, making up amalgam models and constructed the case on models in sections, assembling as in fixed bridgework construction. My preference now is to take an exact impression of the teeth and spaces and cast these shorter cases all in one piece, a cast saddle, base and pin, using the loose pin crown in preference to trying to cast clasp and saddle separately and assembling later as separate units. These small saddles are difficult to handle and there is no necessity or occasion for securing that compressed relation of the saddle and the clasp. We are depending in these cases, upon the clasp. We should depend in these short spans upon the clasp practically altogether for the support of the appliance, placing no dependence upon the saddle—using it only as a means of securing the continuity of the tooth you are supplying—and the ridge. You do it so nicely with some of the loose pin crowns, casting base and pin, securing lingual contour and normal restoration in that way. The shrinkage of these short spans does not amount to enough to make any material difference as to the final fitting of it. In order to make a single casting of the appliance we must have an impression of the teeth and alveolar ridge. So that in order to get the impression we take it preferably in complaster, using two half trays. With the ordinary crown and bridge tray, removing tray and making an incision longitudinally across the occlusal surface of the teeth and down between them, and breaking out the impression in halves, we are almost sure to mutilate the imprpession, just at the point where it is most essential to have accuracy of the impression—the contact and occlusal surface of these teeth.

In order to make sure of an accurate impression of all the parts we want an exact impression of the occlusal surfaces, lingual surfaces, the constriction at the neck of the tooth, the approximal surface next the space, undercuts and all. It is better to take the impression in halves, in two half trays, and to predetermine in the impression the line of fracture, including the occlusal surfaces of the teeth. We can not draw these impressions out buccally and lingually, nor can we draw them occlusally, so that in taking the lingual half of the impression to get the occlusal surfaces of the teeth, and for the lingual surfaces of the teeth, we take a half tray as in the upper chart (indicating) and take the lingual side of the teeth. The tray, as you see here, has a rightangle flange (covering the lingual and occlusal) confining the material on to the occlusal surface. Rather than to have the impression broken and mutilated, we prefer to secure in this half of the tray the impression of the occlusal and lingual surfaces of those teeth. We can not draw it out lingually because of the interference of the lingual cusp of the tooth. Nor can we draw it occlusally because of the interference of the constriction at the neck—but we can draw it occluso-lingually. It is very easy to draw it in that direction without breaking it. We are including the occlusal surfaces of the tooth up to the summit of the cusp—the buccal cusp. We have that all in one piece. The material that is forced out buccally is trimmed back with instruments when it is hard, until we can withdraw the impression lingually.

Having the impression of these surfaces of the teeth, all that is necessary is to take another tray of similar form and secure impression of the buccal surfaces of the teeth. We will usually have to cut the impression back from the buccal side so that the material may be drawn lingually. If the edges of the material break off (an indication that you have not cut back enough), you must trim and straighten up the surfaces so that you will have a smooth, definite line in the lingual half of the impression. Predetermine so that you will have the impression in two positive, clean, definite pieces. Having the impression trimmed up, all that is necessary is to soap the surface or put on some separating medium and take the impression of the buccal half. If we have a short space, and not too far back in the region of the bicuspids or in the first molar region, and we have very high cusps, we may have a deep slot in the inlay for an occlusal rest, or in the natural tooth. We may have difficulty in securing an impression with complaster in such cases by reason of its being very fragile.

Here we can, with good understanding of the use of modeling compound, apply this material in the same way.

This represents, then (indicating), a lingual view of the impression and the two halves of the tray with the impression assembled and waxed together ready for pouring and making a model. I use the term "model," which is not up to the latest terminology. "Cast" is the proper word, but it is easy to confuse it with "casting." So we have our impression as in making models for clasps, using liquid silex as a separating medium, etc. Having the model, we are ready to prepare it for waxing up. Some have not seemed to grasp the idea that we are casting directly on to the model. We are not making models and removing the wax pattern, etc. We cast directly on to the model, using inlay investment compound for the purpose. There is an undercut always to contend with; the approximal surfaces of these teeth, molars and bicuspids form more or less of an undercut. We must eliminate the undercut before making the casting. I want the model to be a reproduction of the teeth and ridge before me. I want to determine exactly where I am going to terminate my surface bearing of the cusp or appliance to that tooth. I want to design the appliance, clasp and saddle according to the conditions in the mouth. If we have teeth that are considerably bell-shaped, slightly tipped, and a pronounced undercut, I resort to the use of stones and disks and relieve or remove any excess contour that will not materially injure the teeth. It simplifies the construction and manipulation of the appliance after it is made. After we have this removed, and the impression, it is necessary to eliminate the undercut. In the chart we show how this is done. If we cast in around the teeth and fill in the undercut, you can not get the appliance on at all. So eliminate it before making up the casting. Fill the undercut in the model, building up the approximating surfaces of the teeth in inlay investment compound again. In order to make sure the inlay investment compound will be held securely in place while heating up the case, etc., we make an undercut in the model—cutting into the teeth on both sides, making a mechanical retention for this additional investment material as used to build up the approximating surface of those teeth. It is necessary to eliminate the undercut, and where we have these short spans, with rigid, solid teeth on both sides, there is no chance to trick them in as where we have clasps on each side of the mouth. Sometimes you have to extend that material around buccally and lingually to eliminate the undercut that interferes with the withdrawal and placement of the appliance. You may say you are leaving a place between the dummy and the natural teeth. So do we. We have a space that corresponds to the amount of undercut, representing the additional investment compound we have put on the model. This is an advantage. It is exactly the amount of space you have in the normal relation of these teeth. It is a removable appliance, protected on the occlusal surfaces so that the excursions of the food will not get into that space. It leaves the interproximal space and gingival margins around those teeth free from irritation and from any impingement of the appliance, so that it does not constitute an objection, but rather a favorable condition. So we wax the clasp, saddle and all as indicated, carrying the wax up to the occlusal surface and making provision for occlusal rests, on bicuspid and molar.

We take advantage of gravity in the casting, which is wise to do, regardless of how you are casting. We have found in these cases of partial denture construction, if we can attach the sprue at some point of the wax pattern so that all the wax of that pattern will be below the point of ingress of the gold into the mold, that it requires less force, as you are working in harmony with the law of gravity, which, I think, is a good thing to do. We do this casting with a tin-can lid, blotting paper stem pad without any trouble at all. It is not a question of the kind of machine you use so much as a question of mastering some definite technique. Know the technique and carefully follow it. I can take the same equipment and follow the same technique, carefully,—and still make 50 per cent. more failures than I would by making a slight change in the means of investing, of placing the wax pattern in the mold, size of the sprue in proportion, etc., and the form of the crucible itself—direction of the flame, quality of heat. I mean it is not so much a question of complicated machinery as it is to know the equipment and to master it. So I believe it is better, regardless of the force you use, whether centrifugal, gas or steam pressure, that the gold be forced in at a point above rather than below the point of ingress into the mold, as explained. We make a complete ring of it.

### UNI-LATERAL CASES

These refer to cases where teeth are out on one side only. There are a certain number of these cases that can be very successfully made by the use of the double cast clasp with the embrasure hook, or using only the cast clasp. There are a few conditions that we must take into consideration in determining the practicability of the unilateral construction. First, we must have good, firm, well-defined, fully absorbed alveolar ridges. This is the first requisite. The next is, good strong teeth to which we can attach our clasps. Another favorable condition is a short bite. With these conditions favorable, these appliances are eminently satisfactory, if properly made. The proper construction of these cases means that we must take the impression of the teeth we are going to clasp, make the two clasps, and it is a good rule to make the saddle as large as you can, covering as much surface of the ridge with large saddles and as few teeth as possible, not trying to supply the full complement of teeth that were lost. Better have one tooth comfortable and useful, minimizing the leverage of clasps, etc. Use as few teeth as possible, increasing the size of the saddle as much as possible. Use two clasps if possible. The most striking cases we have ever made were anchored to the lower cuspid, with conditions favorable, cuspids strong and the ridge as large as my finger—hard and firm—and a short bite. You can cast two clasps at one time, casting to the embrasure grip.

Make the saddles separately, unite and place the saddle in the mouth with clasps in position and take impression on to the saddle. To get the compressed relation is rather difficult. We have been shown how to do this in the textbooks, journals and clinics by using orange wood sticks, etc., that we could place between the antagonizing teeth and saddle and have patient close on it and hold the saddle under pressure while making the impression. We have as little confidence in the patient doing this right as in my and your ability to fill root canals. I want to know definitely that I am getting a given pressure on the saddle and maintain it in making impression of the saddle and the clasp.

I regret that it has not been possible to give you a practical demonstration of the splendid results that can be obtained with this cast clasp, but it has not been possible in the time we have had. I would rather do the work than talk about it. If you will work out the details of the technique and handle the materials as it is intended they should be, you will find these methods a most valuable means of meeting the requirements of today. In supplying partial dentures, I am sincere in my belief that the cast clasp in its various types, is our greatest hope for a more universal means of meeting these requirements. I thank you!

